# Daily health and well‐being in adulthood and old age: The role of adverse childhood experiences

**DOI:** 10.1111/aphw.12637

**Published:** 2024-12-16

**Authors:** Sophie Potter, Emma Bridger, Johanna Drewelies

**Affiliations:** ^1^ Psychology Heriot‐Watt University Edinburgh UK; ^2^ Psychology University of Leicester Leicester UK; ^3^ Psychology Lise Meitner Group for Environmental Neuroscience, Max Planck Institute for Human Development Berlin Germany

**Keywords:** adverse childhood experiences, affective reactivity, coping and adaptation, daily health, daily well‐being, life course

## Abstract

How susceptible our daily affect is to fluctuations in physical health indicates how well we adapt to everyday health challenges. Adverse childhood experiences (ACEs) are known to have a lasting impact on everyday emotion regulation and adaptation across the lifespan, but less is known about whether and how such adversity is linked to the susceptibility of affect to everyday health challenges. This study therefore tested whether ACEs were associated with daily reports of positive and negative affect and examined weather they moderated emotional reactions to daily physical symptoms in adulthood and old age. We used data from the National Study of Daily Experiences 2 (NSDE‐2) in which middle‐aged and older adults (*N* = 2,022; *M*_*age*_ = 56 years; range: 33–84) reported symptoms and affect on eight consecutive evenings. Multi‐level models indicated that cumulative ACEs as well as two individual childhood adversities (i.e. physical and sexual abuse by a parent) were independently associated with exacerbated increases in negative affect on days with more symptoms. Findings add to literature on the role of early adversity for the maintenance of everyday well‐being and highlight the potential importance of such experiences for coping and adaption in the face of daily health challenges across adulthood and old age.

## INTRODUCTION

Physical health is a key risk factor for the short‐ and long‐term maintenance of subjective well‐being (SWB) across adulthood and old age. For example, numerous empirical studies report that poor physical health threatens the maintenance of middle‐aged and older adults' global SWB as well as their in situ regulation of positive and negative emotions (Gana et al., 2013; Katana et al., 2020; Potter et al., 2020; Potter, Gerstorf, et al., 2022; Potter, Röcke, et al., 2022; Schöllgen et al., 2016; Waldinger & Schulz, 2010). Moreover, the strength of daily health and well‐being associations may have harmful consequences given the physiological effects of activated and maintained emotional distress (see Potter, Gerstorf, et al., 2022 and Schöllgen et al., 2016 for a full discussion). Yet, our physical health as well as our ability to process and regulate emotions in adulthood is profoundly shaped by our early life experiences, especially ones that are traumatic (Nusslock & Miller, 2016; Riediger & Bellingtier, 2022). Indeed, adverse childhood experiences (ACEs)—a term used to describe potentially traumatic childhood events that impact later life health and well‐being—are extremely common in the Western world (WHO: Sethi et al., 2018) and have been linked to more frequent physical health challenges (Hughes et al., 2017), fewer coping and adaption resources, and lower daily well‐being (Infurna et al., 2015; Myroniuk et al., 2024) among adults in the general population. Studies also show that ACEs are associated with elevated emotional reactivity to the minor hassles and challenges of daily life (e.g. Cristóbal‐Narváez et al., 2016; Kong, Liu, et al., 2021; Kong, Martire, et al., 2021; Infurna et al., 2015; Poon & Knight, 2012; van Nierop et al., 2018; Weltz et al., 2016), but none have tested these considerations in the context of daily *health* challenges. To address this gap in the literature, this study examined whether and how ACEs moderated emotional reactions in positive affect (PA) and in negatice affect (NA) to day‐to‐day fluctuations in physical health among adults and older adults.

## DAILY HEALTH AND WELL‐BEING ASSOCIATIONS IN ADULTHOOD AND OLD AGE

The experience of physical symptoms in daily life, such as headache, chest pain, and shortness of breath, are common in the general population (Ihlebæk et al., 2002), especially among middle‐aged and older adults. Indeed, normative declines in physical health across midlife and old age may be reflected in more frequent or volatile daily symptoms (e.g. Bruusgaard et al., 2012; Wolff et al., 2012), thereby posing a threat to how well middle‐aged and older adults emotionally respond to and handle everyday health challenges. To illustrate, conceptual accounts emphasize that such ageing‐related challenges may work to undermine the coping and adaption resources otherwise used to maintain emotional well‐being (e.g. Charles, 2010). Correspondingly, there is growing evidence of emotional reactivity to everyday health challenges across adulthood and old age: Micro‐longitudinal studies report that increased day‐to‐day fluctuations in physical symptoms go hand‐in‐hand with increased fluctuations in both positive and negative affect (Katana et al., 2020; Potter, Gerstorf, et al., 2022; Potter, Röcke, et al., 2022; Waldinger & Schulz, 2010).

Lifespan developmental theory has long emphasized that numerous socio‐contextual factors, individual difference characteristics, and early life experiences influence our capacity to handle the challenges of everyday life (Baltes, 1987; Schafer & Ferraro, 2012). Indeed, in recent years, a number of studies have begun examining risk or resilience factors that buffer how well adults and older adults handle physical health challenges, including personality, ego‐resiliency, and social relationships (Bak et al., 2023; Potter et al., 2020; Potter, Röcke, et al., 2022; Waldinger & Schulz, 2010). However, these studies focused on factors or experiences that occur in adulthood or old age (e.g. ageing‐related changes in personality, social support from a long‐term spouse). Taking a full lifespan approach, we move the study of daily health and well‐being dynamics ahead from such reports by examining the role of early adversity for coping and adaption therein.

## DAILY HEALTH AND WELL‐BEING ASSOCIATIONS: THE MODERATING ROLE OF ACEs

The widely used and influential ACEs framework developed by the Centers for Disease Control and Prevention (CDC; Felitti et al., 1998) defines childhood adversity as potentially traumatic childhood experiences related to threat (e.g. physical, emotional, sexual abuse), deprivation (e.g. emotional abuse), and household dysfunction or challenge (e.g. parental substance abuse, parental divorce, financial distress, moving frequently). There are a number of ways in which these ACEs may influence coping and adaption and be associated with emotional reactivity to daily health challenges in later life. To begin with, Constructed Emotions Theory (Barrett, 2017) indicates that exposure to negative experiences in early life influences the development of core emotional processes involved in adult emotional reactivity. For example, early negative events are thought to equip people with a more extensive and easily accessible repertoire of negative emotion concepts, as well as a baseline emotional state biased toward negative valence. Early negative experiences are also thought to influence adult interoception (the brain's perception and interpretation of internal bodily states), which may result in hypervigilance and heightened responsiveness to short‐term changes in physical health (see Maunder et al., 2022). This implies that exposure to early adversity might alter core processes related to the regulation and expression of negative affect. Indeed, these emotional processes have been linked to elevated emotional reactivity and can be detected in adults with a history of ACEs (Gruhn & Compas, 2020; Harms et al., 2019).

Neurobiological perspectives also indicate that ACEs may be associated with elevated emotional reactivity to daily health challenges in both positive and negative affect because of damage to physical and emotional development (see Bronfenbrenner & Evans, 2000). To illustrate, child adversity is often characterized by decreased exposure to language, linguistic complexity, and scaffolded learning environments (Sheridan et al., 2020), which can alter biological set points leading to poorer health outcomes in adulthood (e.g. Galobares et al., 2008) as well as the (under)development of brain regions involved in everyday emotional regulation and reactivity (Teicher & Samson, 2013). Other perspectives hold that ACEs may be linked to elevated emotional reactivity in adulthood via diminished coping skills and stress dysregulation. For example, as with the allostatic load literature, repeated or prolonged toxic stress exposure from ACEs is thought to cause physiological wear‐and‐tear resulting in damage to the emotional system (McEwen, 1998, 2003), with evidence that adults with a history of ACEs emotionally react with lower‐than‐average PA and higher‐than‐average NA to minor hassles and challenges of everyday life (e.g. Infurna et al., 2015).

It is possible that ACEs are associated with adult emotional reactivity to health challenges via these pathways through *cumulative risk* of multiple co‐occurring adversities and/or through the unique circumstances and attributes of specific *adversity types* (for overviews, see Evans et al., 2013 and McLaughlin & Sheridan, 2016). Cumulative risk is based on the idea that childhood adversities that frequently co‐occur cause greater impact to physical and emotional development than singular exposure. Indeed, children who encounter one adversity often experience multiple adversities (McLaughlin & Sheridan, 2016) and studies report dose‐dependent associations among ACEs and daily health and well‐being in later life (e.g. Berhe et al., 2023). Consistent with cumulative risk perspectives, it stands to reasons that experiencing more co‐occurring ACEs more directly or severely alter the core emotional, neurobiological, and physiological processes discussed above. As one example, more co‐occurring ACEs presumably entail more severe and prolonged toxic stress exposure and come with increasingly less enriching environments and are thus more likely to contribute to stress dysregulation and neurobiological processes (respectively) to prompt elevated emotional reactivity. Indeed, although not tested in the context of daily health, a number of studies have reported that cumulative ACEs moderate emotional reactivity to the minor hassles and challenges of daily life, such that adults who have experienced more co‐occurring ACEs report higher levels of NA and/or lower levels of PA on days with more minor stressors (e.g. Infurna et al., 2015; Kong, Liu, et al., 2021; Poon & Knight, 2012).

On the other hand, different adversities may influence emotional development and be associated with later life emotional reactivity via unique pathways or else contribute more, less, or not at all to the cumulative risk pathway. For example, dimensional approaches to child adversity and later life health and well‐being, including the dimensional model of adversity and psychopathology (DMAP: McLaughlin & Sheridan, 2016), emphasize that groups of individual adversities affect emotional development—and later life emotional reactivity—based on their shared features (i.e. threat vs. deprivation). From this perspective, ACEs related to threat (i.e. physical, emotional, sexual abuse) are potentially more likely to be associated with *elevated* emotional reactivity to daily health challenges because these adversities are more closely associated with toxic stress exposure than deprivation‐based adversities (i.e. emotional neglect). Threat‐based adversities are also known to sensitize fear learning processes and reactivity to negative stimuli (Cicchetti & Toth, 2016). For example, threatening situations, such as physical, emotional, or sexual abuse perpetrated by a parent, are thought to influence information processing and emotional functioning leading to an increased awareness of and sensitivity to negative stimuli in everyday life, such as physical symptoms (e.g. Zautra et al., 2002). Consistent with these lines of reasoning, there is mounting evidence that physical, emotional, and sexual abuse are linked to elevated emotional reactivity to minor hassles and challenges of daily life (Weltz et al., 2016), implying that a similar pattern might be detected for health challenges in daily life.

On the other hand, individual adversities related to deprivation (e.g. emotional neglect) are potentially more likely to be associated with *reduced* emotional reactivity to health challenges in daily life. This is because deprivation is associated with a lack of basic human needs which when teamed with less child–parent interactions, inconsistent learning environments, and fewer scaffolded learning opportunities may lead to the underdevelopment of cognitive and emotional processes needed for the experience and regulation of emotions in everyday life (Mikulincer & Shaver, 2010; Sheridan et al., 2020). Correspondingly, childhood deprivation is often associated with infrequent or blunted displays of emotion (Berzenski, 2019), and initial evidence indicates that those with a history of neglect demonstrate below‐average emotional reactivity toward minor hassles and challenges in daily life (Weltz et al., 2016).

It is unclear whether ACEs related to household dysfunction or challenge would be related to adult emotional reactivity. For example, although some of these ACEs, including financial distress, parental divorce, and frequently moving homes, are typically considered stressful by children, these do not necessarily denote *toxic* stress exposure in the same way or degree as abuse‐based adversities. Although no studies to date have examined independent associations among these ACEs and adult emotional reactivity, some studies that have tested their associations with (trait‐report) adult mental health, reporting either no or weak associations (e.g. Atzl et al., 2019).

## PRESENT STUDY

The aim of this study was to extend existing research on daily health and well‐being dynamics and provide a life‐course approach to coping and adaption therein by examining the role of ACEs for everyday health and well‐being among middle‐aged and older adults. To do so, we tested the role of individual and cumulative ACEs for daily positive and negative affect and examined weather they moderated emotional reactions to daily physical symptoms. To do so, we applied multi‐level models to daily data from the National Study of Daily Experiences 2 (NSDE‐2) and retrospective ACEs data from the corresponding Midlife in the United States (MIDUS) studies. We measured ACEs using a scale developed by previous researchers, namely, Danielson and Sanders (2018), with existing MIDUS items to replicate the well‐established and widely used CDC‐Kaiser ACEs scale (Felitti et al., 1998). The scale included individual adversities related to threat (i.e. physical, emotional, sexual abuse), deprivation (i.e. emotional abuse), and household dysfunction or challenge (i.e. parental substance abuse, parental divorce, financial distress, moving frequently). To comprehensively examine multiple theoretical perspectives, we independently tested moderation by individual and cumulative ACEs. A number of previous studies examining individual adversities and later life emotional reactivity have not statistically accounted for their conceptual overlap (e.g. Kong, Martire, et al., 2021; Poon & Knight, 2012), which can obfuscate their independent effects and make it hard to disentangle unique associations (see Evans et al., 2013). We therefore examined independent associations of all individual adversities in a single statistical model.

To begin with, we expected cumulative and individual ACEs to be associated with levels of daily well‐being, specifically lower PA and higher NA per day. In terms of emotional reactivity, based on conceptual accounts of the cumulative impact of ACEs on physical and emotional development (Bronfenbrenner & Evans, 2000; McEwen, 1998, 2003) as well as with evidence of their associations with elevated emotional reactivity to minor hassles and challenges of daily life (e.g. Infurna et al., 2015; Kong, Liu, et al., 2021), it was expected that cumulative ACEs would be associated with *elevated* emotional reactivity to daily health challenges. That is, we expected cumulative ACEs to be associated with above‐average NA and below‐average PA on days with more physical symptoms. Based on dimensional models of child adversity and later life health and well‐being (e.g. McLaughlin & Sheridan, 2016) as well as with initial evidence of differential associations among individual ACEs and adult emotional reactivity (e.g. Weltz et al., 2016), it was expected that adversities related to threat (i.e. physical, emotional, and sexual abuse) would be independently associated with *elevated* emotional reactivity to daily health challenges, while the adversity related to deprivation (i.e. emotional neglect) would be associated with *lower* emotional reactivity to daily health challenges. No hypotheses were made for whether adversities related to household dysfunction/challenge would be associated with emotional reactivity due to a lack of theoretical and empirical evidence.

## METHODS

### Participants and procedure

We used data from 2022 participants (*M*_age_ = 55 years; *SD* = 13.03; range: 33–84 years; 57% female) in the NSDE‐II (2004–2009). We used retrospective ACE measures from the associated MIDUS‐I (1995–1996) and MIDUS‐II (2004–2006) studies (see measurement section for further details). For the NSDE‐II study, participants completed telephone interviews about their daily experiences of affect and physical symptoms across eight consecutive days. Interviews were conducted with *N* = ~20 participants in separate waves throughout 2004 to 2009 (for further details, see Almeida et al., 2009). Respondents provided seven daily interviews on average (*SD* = 0.31) with a completion rate of 92%. Twenty participants were excluded from analyses because of missing data on study variables, resulting in a final analysis sample of 2002. Most participants had completed high school (94%), were White (93%), and were married (72%). Data collection was approved by the Institutional Review Board of the Pennsylvania State University. All participants participated anonymously, voluntarily and gave their informed consent at the beginning of the survey.

### Measures

#### ACEs

Individual items from MIDUS‐I and MIDUS‐II were used by previous researchers—Danielson and Sanders (2018)—to create a measure of ACEs reflective of the original CDC‐Kaiser ACEs scale (CDC, Felitti et al., 1998). The measure includes items from MIDUS‐I and MIDUS‐II to create one overarching measurement tool. A detailed overview of the measure, including its relation to the CDC‐Kaiser ACEs scale, is provided in Table S1.

Numerous items were selected to represent eight types of ACEs corresponding as closely as possible to the CDC ACEs framework: physical abuse perpetrated by a parent, emotional abuse perpetrated by a parent, sexual abuse perpetrated by a parent, emotional neglect by a parent, living with a household member with substance use issues, financial distress, parental divorce, and moving homes frequently. Across all items, participants were asked to report their experiences from when they were a child or teenage. A full list of questions and response formats for items within each category are reported in Table S1. As one example, the following question was used to measure physical abuse: “When you were growing up, how often did your mother/father, or the woman/man who raised you, kick, bit, or hit you with a fist; hit/tried to hit you with something; beat you; choked you; burned or scalded you?” answered on a 4‐point Likert scale (1 = *never*; 2 = *rarely*; 3 = *sometimes*; 4 = *often*). Categories were dichotomized to indicate the presence of that adversity. To illustrate using the example above, participants that responded *often, sometimes, or rarely* to physical abuse by their mother or father were coded as having experienced that abuse. The cumulative risk score was created by summing together the eight categories (α = 0.61; range: 0–8) (as per CDC, Felitti et al., 1998; see also Anda et al., 2006). Individual categories (where “1” indicated the presence of that adversity) were used to examine adversity‐specific associations. Note that some of the items or categories used by Danielson and Sanders (2018) differed slightly from the original CDC measure due to data constraints (i.e. not all adversities were sampled in the MIDUS studies).

#### Daily positive and negative affect

Participants reported how much of the time in the past 24 h they experienced 13 positive emotions (*in good spirits, cheerful, extremely happy, calm and peaceful, satisfied, full of life, close to others, like you belong, enthusiastic, attentive, proud, active, confident*; within‐person α = .78) and negative emotions (*restless or fidgety, nervous, worthless, so sad nothing cheer you up, everything was an effort hopeless, lonely, afraid, jittery, irritable, ashamed, upset, angry, frustrated*; within‐person α = .71) on a 5‐point scale (1 = *none of the time*; 5 = *all of the time*). Items were averaged and summed to create a total within‐person PA score (*M* = 2.71; *SD* = 0.81; Median = 2.04; Mode = 2) and a total within‐person NA score (*M* = 1.11; *SD* = 0.91; Median = 1.12; Mode = 1). As is commonplace in the daily diary literature, emotional reactivity was defined as changes in positive or negative affect on days with a higher number of physical symptoms.

#### Daily physical symptoms

Participants were asked whether (1 = yes; 2 = no)^1^ they had experienced the following 28 physical symptoms that day: *headache, backache, muscle soreness, fatigue, joint pain, muscle weakness, cough, sore throat, fever, chills, other cold or flu symptoms, nausea, allergies, diarrhea, constipation, poor appetite, other stomach problems, chest pain, dizziness, shortness of breath, menstrual related symptoms, hot flashes or flushes, any other physical symptoms*, *skin related symptoms, eye related symptoms, ear related symptoms, teeth related symptom, leg or foot related symptoms*. These were summed into a composite score and averaged within‐persons (*M* = 2.69; *SD* = 1.92; Median = 2.12; Mode = 2).

#### Covariates

Analyses accounted for age (grand‐mean centered), sex (0 = *female*; 1 = *male*), and education (1 = *no school or some grade school*; 12 = *doctoral or other professional degree*) to adjust for sample heterogeneity and because these sociodemographic variables are widely thought to confound links between ACEs and a range of health and well‐being outcomes (see Evans et al., 2013). Other variables, including preexisting health conditions and perceived overall health, are linked to associations among ACEs and health outcomes (Jaen et al., 2023) and have been shown to modulate everyday emotional reactivity to daily health challenges (Potter, Gerstorf, et al., 2022). Analyses therefore additionally accounted for chronic conditions (participants listed the number of chronic conditions they have had in the last 12 months; range: 0–27) and self‐reported health (“In general, would you say your physical health is …,” 1 = *poor*; 2 = *fair*; 3 = *good*; 4 = *very good*; 5 = *excellent*).

### Data analysis

Multi‐level models were used to accommodate data nested within‐persons and across days. PA and NA were modelled separately as outcomes in all models (referred to as *affect* in models below for simplicity). At Level 1, daily affect was modelled as a function of physical symptoms (referred to as *symptoms* in models below, i.e. an estimate of emotional reactivity to daily symptoms). Cumulative versus individual ACEs were tested in separate models. For models analyzing cumulative ACEs, the total ACE score was entered as a Level 2 predictor. Likewise, for models analyzing individual ACEs, all eight adversities were tested as Level 2 predictors. In both models, ACEs were tested along with the same set of covariates. Level 1 was specified as

(1) $${\text{Affect}}_{\mathrm{ti}}=\beta_{\text{0i}}+\beta_{\text{1i}}\;\left({\text{Symptoms}}_{\mathrm{ti}}\right)+e_{\mathrm{ti}}$$

where Affect_ti_ of person *i* at time *t* is a function of a person‐specific intercept (β_0i_), person‐specific coefficients indicating the extent to which daily PA and NA is associated with daily health (β_1i_), and with residual error (e_ti_). Between‐person differences in intercept and emotional reactivity to daily symptoms were modelled as

(2) $$\beta_{\text{0i}}=\gamma_{00}+\gamma_{01}\left({\mathrm{Age}}_{i}\right)+\gamma_{02}\left({\text{Women}}_{i}\right)+\gamma_{03}\left({\text{Education}}_{i}\right)+\gamma_{04}\left(\text{Chronic}\;{\text{Condition}}_{i}\right)+\gamma_{05}\left(\text{Self}-\text{Report}\;{\text{Health}}_{i}\right)+\gamma_{06}\left({\text{ACEs}}_{i}\right)+u_{\text{0i}},$$

(3) $$\beta_{\text{1i}}=\gamma_{10}+\gamma_{11}\left({\text{ACEs}}_{i}\right)$$

where γ_00_ and γ_10_ indicate prototypical levels of positive/negative affect and emotional reactivity to daily symptoms, respectively. Parameters γ_01–06_ indicate the extent to which between‐person differences in affect are related to ACEs and covariates. Parameter γ_11_ indicates the extent to which differences in emotional reactivity to daily symptoms were related to ACEs. Person *i*'s deviation from the intercept is denoted by u_0i_. Models were conducted in SAS 9.4 with incomplete data being treated as missing‐at‐random under full information likelihood (Little & Rubin, 2019).

## RESULTS

Descriptive statistics and intercorrelations are presented in Table 1. Unconditional intercept‐only models showed the between‐person variance (intra‐class correlation) to be 45% for PA, 49% for NA, and 52% for daily symptoms, indicating substantial within‐person variability. At MIDUS‐II, respondents reported an average of two chronic conditions (*M* = 2.44, *SD* = 2.31; range: 0–17) and had 3 + years of college (*M* = 6.85, *SD* = 2.51). Respondents reported an average of at least one ACE (*M* = 1.15, *SD* = 1.37) and reported a physical symptom on 66% of the study days. See Table S2 for the frequency of individual ACEs by demographics and daily symptoms.

**TABLE 1:** Descriptive statistics and intercorrelations among study variables.

	1	2	3	4	5	6	7	8	9	10	11	12	13	14	15	16	17
1 Age	1																
2 Female	.02	1															
3 Education	−.10	−.10	1														
4 Self‐report health	−.16	−.03	.25	1													
5 Chronic condition	.18	.12	−.13	−.41	1												
6 ACE cumulative	−.11	.10	−.14	−.13	.20	1											
7 Parental divorce	−.08	.04	−.13	−.07	.08	.49	1										
8 Parental subs. abuse	−.09	.05	−.10	−.06	.07	.55	.18	1									
9 Sexual abuse	−.11	.20	−.03	−.08	.13	.37	.08	.11	1								
10 Physical abuse	−.05	.01	−.06	−.09	.14	.52	.12	.14	.12	1							
11 Emotional abuse	−.08	.05	−.06	−.07	.16	.60	.13	.20	.14	.63	1						
12 Emotional neglect	−.02	.09	−.06	−.08	.10	.51	.15	.15	.14	.29	.35	1					
13 Moved frequently	.01	.03	−.07	−.09	.09	.48	.20	.10	.06	.10	.10	.08	1				
14 Financial distress	.01	.00	−.08	−.07	.10	.51	.17	.19	.07	.12	.17	.15	.11	1			
15 Positive affect	.17	.00	−.06	.14	−.15	−.13	−.05	−.09	−.11	−.06	−.09	−.09	−.04	−.03	1		
16 Negative affect	−.11	.05	.01	−.11	.17	.09	.04	.05	.11	.04	.09	.07	.03	.03	−.49	1	
17 Daily symptoms	.02	.12	−.09	−.24	.36	.11	.08	.06	.10	.04	.05	.04	.07	.09	−.31	.42	1
*Mean* or %	56.12	57%	6.85	3.51	2.44	1.15	22%	23%	9%	7%	11%	11%	27%	20%	2.71	1.11	2.69
*SD*	13.03		2.51	1.01	2.31	1.31									0.81	0.91	1.92

*Note*: Intercorrelations of *r* = >.08 differ statistically significantly from zero at *p* = .01.

Abbreviations: ACEs, adverse childhood experiences; subs., substance.

### ACEs and daily health and affect

The correlations in Table 1 indicate that reporting more ACEs was associated with more daily physical symptoms (*r* = .11, *p* < .01), lower levels of daily PA (*r* = −.13, *p* < .01), and higher levels of daily NA (*r* = .09, *p* < .01). In a preliminary set of analyses aimed at inspecting descriptive statistics further, we created groups of low versus high levels of ACEs based on a median split of the cumulative ACEs score and used *t*‐test analysis to examine differences in key study variables. Table 2 shows that individuals who reported higher levels of ACEs were, on average, younger, female, had more chronic conditions, and lower self‐reported health but did not differ on levels of education. Moreover, those with higher levels of ACEs reported more daily symptoms, lower levels of daily PA, and higher levels of daily NA (see Table S3, for differences in discrete emotions by high vs. low ACEs). To examine this further, we used odds ratios to test whether more cumulative ACEs were associated with the likelihood of reporting daily symptoms. We found associations between cumulative ACEs and likelihood of reporting a symptom per day (Odds Ratio = 1.21, 95% confidence interval: 1.13, 1.31), indicating that individuals with one additional ACE were 21% more likely to report a physical symptom on a given day compared to those with fewer ACEs.

**TABLE 2:** Examining mean‐level differences on key study variables based on low versus high levels of adverse childhood experiences.

ACE	Low ACEs (*N* = 1536)		High ACEs (*N* = 466)	
	*M*	*SD*	*M*	*SD*
Age	46.59^a^	13.19	45.17	11.74
Sex	1.49^a^	0.50	1.61	0.49
Education	6.79	2.49	6.79	2.47
Chronic conditions	2.32^a^	2.45	2.90	2.74
Self‐reported health	3.54^a^	1.00	3.50	0.95
Daily physical symptoms	1.80^a^	2.18	2.09	2.29
Daily positive affect	2.78^a^	0.78	2.60	0.81
Daily negative affect	0.19^a^	0.32	0.22	0.33

*Note*: Subscripts indicate a statistically significant difference.

Abbreviation: ACE, adverse childhood experience.

### Emotional reactivity to daily health challenges

As can be seen in Table 3, consistent with previous literature (e.g. Infurna et al., 2015; Myroniuk et al., 2024), those with more cumulative ACEs reported lower levels of PA per day (β = −0.05; *p* = .0002) and higher levels of NA per day (β = 0.01; *p* = .0001). As can be seen in Table 4, we also found lower levels of daily PA among those who lived with a parent with substance abuse issues growing up (β = −0.11; *p* = .001), as well as among those who had experienced childhood sexual abuse (β = −0.13; *p* = .004) and emotional neglect (β = −0.14; *p* = .001). Higher levels of daily NA were also found among those exposed to childhood sexual abuse (β = 0.05; *p* = .0001) and emotional abuse (β = 0.05; *p* = .0002). Individual adversities related to household challenge (financial distress, moved homes frequently, and parental divorce) were unrelated to daily PA and NA.

**TABLE 3:** Multi‐level models examining daily health and affect associations and their moderation by cumulative adverse childhood experiences (ACEs).

	Positive affect			Negative affect		
	Est.	*CI*_*95*_		Est.	*CI*_*95*_	
		Lower	Upper		Lower	Upper
Intercept	2.644*	2.533	2.755	0.181*	0.144	0.217
Age	0.011*	0.008	0.014	−0.003*	−0.004	−0.01
Sex	0.046	−0.020	0.114	0.007	−0.015	0.029
Education	−0.029*	−0.043	−0.015	0.003	−0.002	0.008
Health self‐report	0.105*	0.064	0.145	0.014*	−0.027	0.001
Chronic conditions	−0.040*	−0.057	−0.025	0.022*	0.016	0.026
ACEs cumulative	−0.051*	−0.075	−0.027	0.011*	0.003	0.019
Daily symptoms	−0.058*	−0.064	−0.052	0.040*	0.030	0.050
Daily symptoms*cum. ACEs	0.004	−0.000	0.007	0.004*	0.002	0.006
Variance intercept	0.414	0.359	0.425	0.041	0.032	0.052
Residual variance	0.138	0.113	0.145	0.045	0.031	0.054

Abbreviations: Cum., Cumulative; Est., Estimate.

* *p* < .05.

**TABLE 4:** Multi‐level models examining daily health and affect associations and their moderation by type of adverse childhood experience (ACE).

	Positive affect			Negative affect		
	Est.	*CI*_*95*_		Est.	*CI*_*95*_	
		Lower	Upper		Lower	Upper
Intercept	2.616*	2.50	2.729	0.187*	0.149	0.224
Age	0.010*	0.008	0.013	−0.003*	−0.004	−0.002
Sex	0.062	−0.006	0.131	0.003	−0.020	0.026
Education	−0.029*	−0.043	−0.015	0.002	−0.002	0.007
Trait‐report health	0.107*	0.067	0.147	−0.014*	−0.028	−0.001
Chronic conditions	−0.040*	−0.056	−0.025	0.021*	0.016	0.027
Daily symptom	−0.059*	−0.065	−0.053	0.040*	0.037	0.044
Parental divorce	0.014	−0.082	0.111	−0.018	−0.050	0.014
Parent substance abuse	−0.109*	−0.194	−0.025	0.022	−0.006	0.050
Sexual abuse	−0.133*	−0.252	−0.014	0.048*	0.008	0.087
Physical abuse	0.071	−0.103	0.246	−0.053	−0.111	0.005
Emotional abuse	−0.089	−0.231	0.053	0.049*	0.001	0.096
Emotional neglect	−0.141*	−0.259	−0.023	0.014	−0.025	0.053
Moved frequently	−0.038	−0.115	0.038	0.016	−0.010	0.041
Financial distress	0.004	−0.084	0.091	0.007	−0.022	0.036
Daily symptom*sexual abuse	–	–	–	0.012*	0.002	0.022
Daily symptom*physical abuse	–	–	–	0.016*	0.001	0.032
Variance intercept	0.412*	0.381	0.440	0.042	0.037	0.045
Residual variance	0.139*	0.128	0.144	0.043	0.041	0.044

Abbreviation: Est., Estimate.

* *p* < .05.

As with existing research, we also found evidence of emotional reactivity to daily symptoms (i.e. within‐person health–affect associations) across all sets of analyses (see Tables 3 and 4). More specifically, reporting a higher number of physical symptoms per day was independently associated with lower‐than‐average daily PA and higher‐than‐average daily NA. Most important for our research question, we found evidence for the moderating role of individual and cumulative ACEs for such emotional reactivity in NA (but not PA) to daily symptoms.

#### Moderation by individual and cumulative ACEs

**FIGURE 1:**
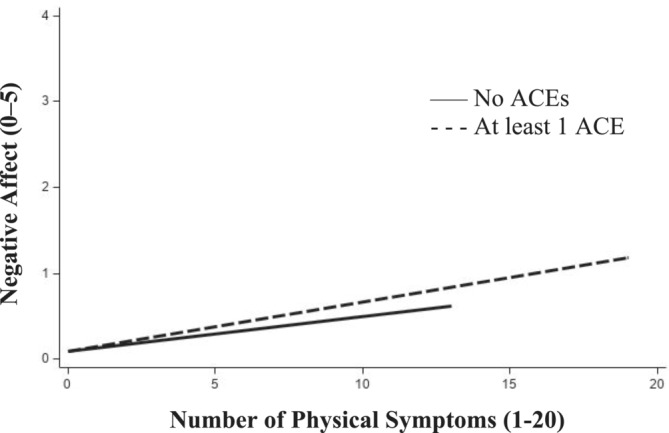
Emotional reactivity to daily physical symptoms: moderation by cumulative adverse childhood experiences (ACEs). Note. On days when participants experienced more physical symptoms than usual, they also reported higher levels of negative affect. Levels of negative affect were higher for those who had experienced at least 1 adverse childhood experience (dashed‐lined) compared to those who had not (solid line).

Results for moderation by cumulative ACEs can be found in Table 3. As illustrated in Figure 1, cumulative ACEs were associated with elevated emotional reactivity in NA toward daily symptoms, with exacerbated increases in NA detected on days with more physical symptoms (β = 0.004; *p* = .0001). Cumulative ACEs were unrelated to emotional reactivity in PA toward daily symptoms.

**FIGURE 2:**
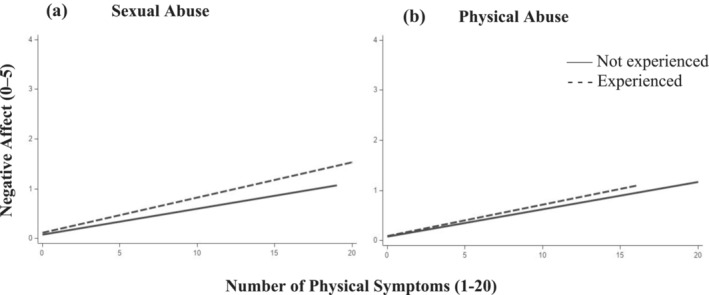
Emotional reactivity to daily physical symptoms: moderation by individual adverse childhood experiences. Note. On days when participants experienced more physical symptoms than usual, they also reported higher levels of negative affect. Levels of negative affect were higher for those who had experienced sexual abuse as a child (Plot A, dashed‐lined) and for those who had experienced physical abuse as a child (Plot B, dashed‐lined) compared to those without (solid line).

Results for moderation by individual ACEs can be found in Table 4. Consistent with expectations, a number of adversities related to threat were associated with elevated emotional relativity in NA toward daily symptoms. More precisely, sexual abuse (β = 0.01; *p* = .004) and physical abuse (β = 0.02; *p* = .002) were associated with exacerbated increases in NA on days with physical symptoms. These associations are illustrated in Figure 2, Plot A (for sexual abuse) and Plot B (for physical abuse). The adversity related to deprivation (i.e. emotional neglect) and the adversities related to household dysfunction and/or challenge (i.e. parental substance abuse, parental divorce, financial distress, frequently moving home) were unrelated to emotional reactivity to daily symptoms.

Note that we additionally accounted for variables that have previously shown associations with daily health and affect, namely, daily stressors (e.g. Kong, Liu, et al., 2021) and trait‐report neuroticism (e.g. Potter, Gerstorf, et al., 2022), but our findings did not change with their inclusion. We therefore removed these variables from analyses, following recommended good practice (Grimm et al., 2016).

## DISCUSSION

This study aimed to substantiate and extend existing research on daily dynamics in health and well‐being and provide a life‐course approach to coping and adaption in the face of health challenges. To do so, we tested the role of individual and cumulative ACEs for daily positive and negative affect and examined weather they moderated emotional reactions to daily physical symptoms among middle‐aged and older adults. Consistent with prior research, we found that cumulative ACEs and individual adversities related to threat and deprivation were independently associated with lower daily PA and higher daily NA (e.g. Infurna et al., 2015; Myroniuk et al., 2024). Moreover, evidence arose for the moderating role of cumulative and individual ACEs for emotional reactivity in NA (but not PA) toward daily health challenges. Specifically, experiencing (1) more cumulative ACEs, and (2) individual adversities characterized by threat (i.e. physical and sexual abuse) were independently associated with elevated emotional reactivity to daily physical symptoms (i.e. above‐average increases in NA on days with more symptoms). These findings substantiate and extend previous research on daily health and well‐being in several important ways. First, they contribute to literature on early life experiences and later life health and well‐being by demonstrating that prevalent childhood adversities are associated with a range of everyday health and well‐being experiences in nonclinical adult populations. Second, they substantiate existing work on coping and adaption in the face of everyday health challenges (e.g. Katana et al., 2020; Potter, Gerstorf, et al., 2022; Waldinger & Schulz, 2010) and extend such work by providing first evidence that early adversity may exacerbate emotional reactions to daily symptoms among adults and older adults. Third, they provide initial insight on the potential pathways linking early adversity to later life health and well‐being dynamics and in doing so provide tentative support for the view that more co‐occurring adversities, and/or exposure to individual adversities characterized by threat (but not deprivation or household challenge/dysfunction), are potentially important for emotional reactivity.

### Emotional reactivity to daily health challenges in adulthood and old age

Evidence of emotional reactivity to daily symptoms (i.e. within‐person daily health and affect associations) corroborates growing evidence of the importance of everyday health challenges for the short‐ and long‐term maintenance of SWB (e.g. Katana et al., 2020; Potter, Gerstorf, et al., 2022; Potter, Röcke, et al., 2022; Waldinger & Schulz, 2010). Such findings underscore the relevance of physical health for individual differences in how well we handle ageing‐related challenges in everyday life and in doing so illustrate conceptual accounts on coping and adaption across adulthood and old age (e.g. Charles, 2010).

#### The moderating role of ACEs

Our finding that ACEs were associated with elevated emotional reactivity to daily symptoms provides first evidence to suggest that early adversity is associated with how well we handle prevalent daily health challenges across adulthood and old age. These findings are consistent with broad principles from lifespan developmental theory that experiences from all stages of life are important for coping and adaption (Baltes, 1987). Findings also extend lifespan research on risk and resilience factors that buffer the links between daily health and well‐being. In particular, these results provide first evidence that factors beyond those experienced in midlife and old age (e.g. in situ social support: Potter, Röcke, et al., 2022) dampen emotional reactivity to daily health challenges.

Importantly, very little evidence arose for associations between early adversity and emotional reactivity in PA, suggesting that early adversity is more directly relevant for negative emotional processes in the context of daily health challenges. These findings may therefore be aligned with ideas from Constructed Emotion Theory that exposure to negative events in early life is linked to *negative* emotion concepts and *negative* valence (Barrett, 2017). To provide a more nuanced picture of the links between ACEs and affective well‐being in later life, it might be useful to examine emotional reactivity with discrete emotions differing in valence and arousal. Note also that findings are based on middle‐aged and *young*‐old adults and should thus be substantiated in more advanced old age. It is possible, for example, that increasing age represents a testing‐the‐limits situation that overwhelms the psychological system to further exacerbate the role of early adversity. On the other hand, age‐related improvements in coping and adaptation typically reported with increasing age (see Potter, Gerstorf, et al., 2022; Potter, Röcke, et al., 2022) might dampen the role of early adversity.

Findings on the moderating role of individual versus cumulative ACEs address conceptual accounts of the links between early adversity and everyday emotional experiences in adulthood (e.g.  Sheridan et al., 2020) and in doing so provide initial insight into the potential pathways linking early adversity to later life health and well‐being dynamics. In particular, results support cumulative *and* dimensional pathways (e.g. Evans et al., 2013; McLaughlin & Sheridan, 2016). To illustrate, evidence that cumulative ACEs were associated with elevated emotional reactivity to daily health challenges supports the idea that physical and emotional development is impacted through biological change and physiological wear‐and‐tear from prolonged exposure to unstimulating environments and toxic stress from co‐occurring adversities (Bronfenbrenner & Evans, 2000; McEwen, 1998, 2003). Even if different ACEs do not contribute equally to cumulative risk, it appears to be the experiencing of these together, regardless of type, that is important for daily dynamics in health and well‐being. At the same time, evidence that individual ACEs characterized by threat, but not those characterized by deprivation or household challenge/dysfunction, were associated with elevated emotional reactivity supports dimensional models of early adversity and later life health and well‐being (DMAP: McLaughlin & Sheridan, 2016). To illustrate, these findings support the idea that different adversities share attributes (i.e. threat vs. deprivation) that have distinct consequences for emotional development (McLaughlin & Sheridan, 2016). These findings not only contribute to growing evidence of differential adversity‐specific associations with later life health and well‐being dynamics (e.g. Weltz et al., 2016). They also imply that different adversities contribute differently—or not at all—to cumulative risk and thus migh not all be risk factors for health and well‐being dynamics. This is important because not everyone who experiences one adversity will necessarily experience another.

It is unclear why deprivation—namely, emotional neglect—was unrelated to emotional reactivity to daily health challenges given the links between child emotional neglect and inhibited emotional displays in adulthood (e.g. Berzenski, 2019). Findings are, however, consistent with dimensional models suggesting that threat‐based adversities (i.e. physical abuse) are more closely associated with emotional processing, whereas deprivation‐based adversities are more closely associated cognitive, rather than affective, processes (e.g. DMAP: McLaughlin & Sheridan, 2016). Given that the impact of adversity on the development of emotional systems is thought to depend on developmental timing (Dunn et al., 2018), another possibility is that participants experienced emotional neglect outside potentially sensitive periods of emotional development not captured by the MIDUS measure. For example, there is evidence that emotional neglect is particularly damaging to emotion regulation during very early infancy (Eilert & Buchheim, 2023), which may not have been recalled or reported by participants in this study. Very early experiences of neglect may therefore not be represented in the data, which could have attenuated associations. Although adversity outside these early periods can still significantly impact emotional development, it would be informative to substantiate results in data that is inclusive of these very early experiences by, for example, using objective measures (e.g. court case documents, adoption/foster records) and/or measures that track the timing, severity, and chronology of ACEs. It is also important to note that results on deprivation‐related ACEs were based only on *emotional* neglect, which is limiting as other types of neglect, namely, *physical* neglect, have been linked to physical and emotional outcomes in adulthood (e.g. Grummitt et al., 2022).

It is unsurprising that household dysfunction/challenge was unrelated to emotional reactivity (beyond potentially contributing to cumulative risk) given the lack of theoretical and empirical evidence. Indeed, dimensional approaches typically do not include such adversities in models of the links between ACEs and health and well‐being outcomes (McLaughlin & Sheridan, 2016) and prior studies report weak (or no) associations between these adversities and adult mental health (e.g. Atzl et al., 2019).

Taken together, findings might be relevant to clinical and developmental psychopathology research by underscoring the role of early adversity in nonclinical populations, as a deeper understanding of such can inform theories on the ontogenesis of health and well‐being processes and can help to identify what constitutes (dys/)functional emotional development (e.g. Cicchetti & Ng, 2014; Myroniuk et al., 2024). Findings are also relevant to the stress literature as they provide initial evidence to suggest that cumulative and individual adversities are relevant to emotional reactivity to daily challenges beyond stressors (e.g. Kong, Liu, et al., 2021; Poon & Knight, 2012). Finally, results imply that trauma‐informed approaches to healthcare should extend beyond mental health to the intersection of physical health and well‐being (see Saunders et al., 2023). Evidence should, however, be substantiated in heterogenous samples before firm conclusions can be made.

### Limitations

The retrospective measure of daily health and affect could be limited by recall bias. For example, the well‐documented *positivity effect* can result in an age‐related tendency to underreport health problems (Mather & Carstensen, 2005) and more positively appraise emotions (Charles et al., 2016). While the use of daily assessment is thought to mitigate this effect, it might nonetheless be useful to substantiate results (1) from moment‐to‐moment and (2) with nonverbal or objective indicators of daily health challenges (e.g. performance‐based grip strength). Relatedly, the daily measurements of health and affect were all based on self‐report which could artificially inflated their associations due to shared method variance. It would also be instructive to explore daily health and well‐being dynamics with indicators of health that differ in manageability and severity as more severe health challenges presumably overwhelm coping and adaption to a greater degree.

Although the operationalization of ACEs available in MIDUS corresponds to the well‐established and widely used CDC‐Kaiser scale, it is limited in a number of important ways. Firstly, it omitted some adversities included in the original CDC measure (Felitti et al., 1998), such as physical neglect (see Table S1, for an overview). In addition, although the operationalization of ACEs is considered comprehensive and well‐standardized (Danielson & Sanders, 2018), it did not include potentially relevant adversities, such as physical/emotional abuse perpetrated by a sibling, and so only provides a limited picture of associations among early adversity and adult health and well‐being dynamics. It would therefore be informative to update the MIDUS ACEs scale with adversity data that includes a wider range of perpetrators. As touched upon above, the measurement of ACEs did not include information on timing nor did it include information on severity or duration, though more severe or long‐lasting adversities presumably impact physical and emotional development to a greater degree (e.g. Evans et al., 2013). As discussed above, the ACEs measure is likely subject to recall error making it important to substantiate findings with objective measures (see Böckerman et al., 2023, for an example of objective measures used in research on ACEs and mental health outcomes). Nonetheless, studies often report good‐to‐excellent test–retest reliability (e.g. Yancura & Aldwin, 2009). Furthermore, it is thought that the recollection of early life adversity matters most, with evidence that emotional functioning does not significantly differ between those who reported ACEs versus those who did not despite having experienced childhood adversity, as identified in legal cases (Danese & Widom, 2020). It is also practical to use retrospective ratings as up to 56% adults who report early adversity do not have prospective measures (e.g. court case).

The data structure and analysis used in the present study can only determine whether “good”/“bad” days (in terms of above‐/below‐average PA and NA) are associated with “good”/“bad” days (in terms of above‐/below‐average symptoms), which presupposes a temporal ordering implying causation of health → affect. Because daily health–affect associations are potentially bidirectional (see Katana et al., 2020), it is important for future research to test associations with a measurement approach that can test multiple causal predictions (e.g. the Bivariate Dual Change Model: McArdle & Hamagami, 2001). Note also that although the term emotional *reactivity* has been criticized on similar grounds (e.g. Stawski et al., 2019), we used this terminology to be consistent with, and build upon, extant research (e.g. Infurna et al., 2015).

Another important consideration is that findings on the moderating role of ACEs for later life health and well‐being links are entirely correlational and only provide limited insight on a complex multiply determined association. Although we accounted for a number of relevant covariates, the correlational nature of the design cannot establish causality and rule out relevant confounding variables, such as important genetic influences (see Baldwin et al., 2023; von Stumm, 2024) and systematic injustices and inequality (for a full discussion on the issue of confounding variables, see Jaen et al., 2023). The latter is especially limiting because people from different racial, ethnic, or socioeconomic backgrounds experience different types and levels of early adversity. For example, marginalized groups are known to face more systemic barriers, discrimination, and chronic stressors, which could strengthen associations among ACEs and emotional functioning. Likewise, education and socioeconomic status are associated with access to resources and healthcare that not only intertwine with life‐course trajectories in health but may also be associated with the management of health challenges. The MIDUS sample did not represent those most likely to suffer from these systematic injustices and inequality. Specifically, higher retention rates for MIDUS‐II were found among respondents who were White, female, and married, as well as those with better self‐reported health and higher levels of education (see Ryff et al., 2015). And although MIDUS‐I, MIDUS‐II, and NSDE‐II had similar distributions for age and marital status, the NSDE‐II subsample included more woman and highly educated participants as well as fewer minority participants. However, a positively selected sample may represent a conservative test of our hypothesis: If associations are observed in a sample that may have experienced lower levels of child adversity, it stands to reason that the findings could be even more pronounced in less positively selected samples. It is therefore strongly recommended that findings are substantiated in more diverse and representative samples.

### Conclusion

The results of this study demonstrate that early adversity is associated with emotional reactivity to daily health challenges among middle‐aged and older adults. In particular, results showed that cumulative ACEs and/or individual adversities characterized by threat—specifically physical and sexual abuse—were independently associated with elevated emotional reactivity to daily health challenges (i.e. exacerbated increases in NA on days with more symptoms). Findings substantiate and extend existing research on adult health and well‐being dynamics by providing (1) a life‐course perspective on risk and/or resilience factors to coping and adaption in the face of everyday health challenges, (2) first evidence suggesting that the role of child adversity for adult emotional experiences extends beyond stress‐reactivity, and (3) tentative insight on the differential pathways linking early adversity to adult health and well‐being. Taken together, findings underscore the importance of early adversity and provide impetus for future research to consider such experiences when examining daily health and well‐being dynamics across adulthood and old age.

## CONFLICT OF INTEREST STATEMENT

The author(s) declare no conflicts of interest.

## ETHICS STATEMENT

The MIDUS studies were reviewed and approved by the Education and Social/Behavioral Sciences and the Health Sciences IRBs at the University of Wisconsin–Madison. All methods were performed in accordance with the relevant guidelines and regulations.

## Supporting information


**Table S1.** Measurement details for the Adverse Childhood Experience measurement tool.
**Table S2**. Frequency of ACEs by demographics and daily physical symptoms among NSDE‐II participants.
**Table S3**. Examining differences in mean levels of discrete emotions based on low versus high levels of adverse childhood experiences.

## Data Availability

Data and documentation for all MIDUS projects are open access and available to download (https://www.icpsr.umich.edu/web/ICPSR/series/203; https://midus.colectica.org). Further details on statistical analyses and analytic methods are available on request from the first author. This study was not preregistered.

## ENDNOTE

1 Recoded to yes = 1 and no = 0 for analyses.
