# Supplementary material for: Daily health and well‐being in adulthood and old age: The role of adverse childhood experiences
Source: Appl Psychol Health Well Being. 2024 Dec 16;17(1):e12637. doi: 10.1111/aphw.12637 (PMC11649961; doi:10.1111/aphw.12637)
Supplement: Supplementary file 1 — Table S1. Measurement details for the Adverse Childhood Experience measurement tool. Table S2. Frequency of ACEs by demographics and daily physical symptoms among NSDE‐II participants. Table S3. Examining differences in mean levels of discrete emotions based on low versus high levels of adverse childhood experiences. [file APHW-17-0-s001.docx]

**ONLINE SUPPLEMENTATY MATERIAL**

**Daily Health and Well-Being in Adulthood and Old Age:**

**The Role of Adverse Childhood Experiences**

**Table S1.** Measurement details for the Adverse Childhood Experience measurement tool

| **Variable** | **MIDUS item** | **Original CDC ACE item** | **Rationale in Danielson & Sanders (2018)** |
| --- | --- | --- | --- |
| Parental Divorce | “Did you live with both of your biological parents up until you were 16?” Response: Yes, **No** (22.4%) | Parents separated / divorced (Response: ***Yes***) | Because it reflects disrupted child-adult relationships and strain in the household that could contribute to toxic stress. This variable also comprises other circumstances (i.e., death of a parent, separation, divorce, adoption). |
| Moved home frequently | Moved 3 times or more (14.4%) | N/A | Can cause/reflect stress in the house (Oishi & Schmmack, 2010) |
| Parental Substance abuse in the home | Check list of experiences. “Experiences you have had as a child or teenager. One or both parents drank so often it caused problems.” (Response: ***Checked,*** 13.3%) OR “One or both parents took drugs so often it caused problems” (Response: ***Checked***, 0.6%) OR “When you were growing up, that is during your first 16 years, did you live with anyone who was a problem drinker or alcoholic?” (Response: **Yes**, 20.6%*,* No) | Live with anyone who was a problem drinker or alcoholic?  Live with anyone who used street drugs?  (Response: ***Yes*** to either) | Consistent with original CDC measure |
| Financial distress | “Experiences you have had as a child or teenager: Father or mother did not have a job when they wanted to be working.” (Response: **Checked**, 8.1%) OR “During your childhood and adolescence, was there ever a period of six months or more when your family was on welfare or ADC?” (Response: **Yes**, 7.0%, No) OR “Thinking back to your family's financial situation when you were growing up, was your family better off or worse off financially than the average family was at that time?”. Response: A lot better off (3.0%), Somewhat better off (10.6%), A little better off (12.1%), Same as average family, A little worse off, **Somewhat worse off**, **A lot worse off)** | N/A | MIDUS variables existed that captured other experiences that could contribute to toxic stress in a household. Financial distress can cause or reflect significant stress in the household. (e.g., Gruenewald et al., 2011; Schafer & Ferraro, 2012; Schilling et al., 2007) |
| Sexual abuse | “Experiences you have had as a child or teenager: Sexually assaulted (e.g., forced sexual intercourse or other unwanted sexual contact).” (Response: **Checked**, 7.4%) | Did an adult or person at least 5 years older ever (1) touch/fondle you in a sexual way (2) have you touch their body in a sexual way (3) attempt oral, anal, or vaginal intercourse with you? (4) actually have oral, anal, or vaginal intercourse with you? (Response: ***Yes*** to one or more) | Consistent with original CDC measure |
| Emotional abuse | “When you were growing up, how often did (1) your mother, or the woman who raised you, (2) your father, or the man who raised you, insulted you or swore at you; sulked or refused to talk to you; stomped out of the room; did or said something to spite you; threatened to hit you; smashed or kicked something out of anger?” Response: **Often** (4.8, 6.3%)*,* ***Sometimes*** (14.5, 16.5%), Rarely (23.4, 24.0%), Never (41.6, 34.5%) to either mother/father | “How often did a parent, stepparent, or adult living in your home ever swear at you, insult you, or put you down?” and “How often did a parent, stepparent, or adult living in your home act in a way that made you afraid that you might be physically hurt?” (Response: Never, Once, Twice, Sometimes, ***Often***, ***Very Often***) | Consistent with original CDC measure |
| Physical abuse | “When you were growing up, how often did your (1) mother, or the woman who raised you, (2) father, or the man who raised you, pushed, grabbed, or shoved you; slapped you; threw something at you?”. Response: **Often** (2.9, 3.5%) ***Sometimes*** (12.2, 12.0%), Rarely (26.8, 24.9%), Never (43.7, 41.8%) to either mother/father, OR “When you were growing up, how often did your (1) mother, or the woman who raised you, (2) father, or the man who raised you, kick, bit, or hit you with a fist; hit/tried to hit you with something; beat you; choked you; burned or scalded you?”. Response: **Often** (1.2, 1.8%), **Sometimes** (4.4, 5.4%), **Rarely** (7.1, 8.4%), Never (72.3, 65.9%) to either mother/father | “Did a parent or other adult in the household… (1) push, grab, shove, or slap you? (2) hit you so hard that you had marks or were injured” (Response: Never, Once, Twice, Sometimes, ***Often***, ***Very Often***) | Consistent with original CDC measure |
| Emotional neglect | Parental Affection Scale (Ryff et al., 2015). 7 items from maternal affection scale; 7 items from paternal affection scale (i.e., rating of your relationship, understood problems and worries, could confide in about things that were bothering you, gave you love and affection, gave you time and attention, taught you about life) (Response: scores of **2.00** **or** **less**, 8.8%) | “You knew there was someone to take care of you and protect you?” OR “There was someone in your family who helped you feel important or special?” OR “You felt loved?” (Response: ***Never True***, ***Rarely True***, Often True, Very Often True) | Consistent with original CDC measure |

Note. Percentage of participant responses are provided in brackets. For physical and emotional abuse, the first percentage refers to the abuse perpetrated by the mother and the second refers to abuse perpetrated by the father.

**Table S2.** Frequencies of Individual Items of ACE Variables

**Table S2**. Frequency of ACEs by demographics and daily physical symptoms among NSDE-II participants

| ACE | No. of ACEs | Female | Physical health |
| --- | --- | --- | --- |
| Any ACE | 59.82 | 35.06 | 18.33 |
| 1 Divorce | 19.82 | 12.38 | 8.16 |
| 2 Parent substance abuse | 21.84 | 13.15 | 8.78 |
| 3 Sexual abuse | 9.68 | 8.45 | 5.08 |
| 4 Physical abuse | 6.38 | 3.87 | 2.94 |
| 5 Emotional abuse | 11.13 | 6.96 | 5.22 |
| 6 Emotional neglect | 11.27 | 8.32 | 4.96 |
| 7 Moved frequently | 26.98 | 15.14 | 11.09 |
| 8 Financial distress | 19.64 | 10.75 | 7.78 |

*Note*. % of ACEs: 0 = 40.18; 1 = 30.01; 2 = 14.72; 3 = 7.68; 4 = 3.90; 5 = 1.86; 6 = 1.09; 7 = 0.38; 8 = 0.

**Table S3**. Examining differences in mean levels of discrete emotions based on low versus high levels of adverse childhood experiences

|  | Low ACEs (*N* = 1,556) | | High ACEs (*N* = 466) | |
| --- | --- | --- | --- | --- |
| ACE | *M* | *SD* | *M* | *SD* |
| Restless | 0.38 | 0.75 | 0.40 | 0.37 |
| Nervous | 0.25^a^ | 0.62 | 0.30 | 0.67 |
| Worthless | 0.07 | 0.37 | 0.08 | 0.38 |
| Sad | 0.06 | 0.36 | 0.07 | 0.35 |
| Effortful | 0.28 | 0.78 | 0.28 | 0.73 |
| Hopeless | 0.06 | 0.38 | 0.08 | 0.38 |
| Lonely | 0.13^a^ | 0.51 | 0.16 | 0.54 |
| Afraid | 0.05^a^ | 0.31 | 0.07 | 0.33 |
| Jittery | 0.13 | 0.47 | 0.15 | 0.13 |
| Irritable | 0.27^a^ | 0.64 | 0.34 | 0.68 |
| Ashamed | 0.03^a^ | 0.24 | 0.05 | 0.27 |
| Upset | 0.26^a^ | 0.63 | 0.34 | 0.70 |
| Angry | 0.19^a^ | 0.54 | 0.23 | 0.58 |
| Frustrated | 0.44^a^ | 0.75 | 0.54 | 0.80 |
| In good spirits | 3.02^a^ | 0.84 | 2.91 | 0.87 |
| Cheerful | 2.87^a^ | 0.94 | 2.68 | 1.03 |
| Happy | 2.05 | 1.32 | 1.66 | 1.34 |
| Peaceful | 2.83^a^ | 0.95 | 2.66 | 1.03 |
| Satisfied | 2.93^a^ | 0.91 | 2.79 | 0.99 |
| Full of life | 2.69^a^ | 1.12 | 2.45 | 2.41 |
| Close to others | 2.80^a^ | 1.04 | 2.65 | 1.09 |
| Belonging | 3.12^a^ | 0.91 | 2.98 | 0.97 |
| Enthusiastic | 2.59^a^ | 1.10 | 2.39 | 1.17 |
| Attentive | 2.89 | 0.91 | 2.81 | 0.93 |
| Proud | 2.57^a^ | 1.20 | 2.31 | 1.27 |
| Active | 2.75^a^ | 1.04 | 2.63 | 1.08 |
| Confident | 3.02^a^ | 0.86 | 2.91 | 0.90 |

***Note.*** Subscripts indicate a statistically significant difference.
